# Reduced Placental Telomere Length during Pregnancies Complicated by Intrauterine Growth Restriction

**DOI:** 10.1371/journal.pone.0054013

**Published:** 2013-01-11

**Authors:** Jérôme Toutain, Martina Prochazkova-Carlotti, David Cappellen, Ana Jarne, Edith Chevret, Jacky Ferrer, Yamina Idrissi, Fanny Pelluard, Dominique Carles, Brigitte Maugey-Laulon, Didier Lacombe, Jacques Horovitz, Jean-Philippe Merlio, Robert Saura

**Affiliations:** 1 EA 2406 Histologie et pathologie moléculaire des tumeurs, Université Bordeaux Segalen, Bordeaux, France; 2 Service de génétique médicale, CHU de Bordeaux, Bordeaux, France; 3 Service de biologie des tumeurs, CHU de Bordeaux, Pessac, France; 4 INSERM U897 Equipe de biostatistique, ISPED, Université Bordeaux Segalen, Bordeaux, France; 5 Service de pathologie, CHU de Bordeaux, Bordeaux, France; 6 Service d’imagerie anté-natale, de l’enfant et de la femme, CHU de Bordeaux, Bordeaux, France; 7 EA 4576 Maladies rares : génétique et métabolisme, Université Bordeaux Segalen, Bordeaux, France; 8 Service de gynécologie-obstétrique et médecine fœtale, CHU de Bordeaux, Bordeaux, France; University of Connecticut, USA, United States of America

## Abstract

**Objectives:**

Recent studies have shown that telomere length was significantly reduced in placentas collected at delivery from pregnancies complicated by intrauterine growth restriction secondary to placental insufficiency. Placental telomere length measurement during ongoing pregnancies complicated by intrauterine growth restriction has never been reported. This was the main objective of our study.

**Methods:**

In our center, late chorionic villus samplings were performed between 18 and 37 weeks of amenorrhea in 24 subjects with severe intrauterine growth restriction (cases) and in 28 subjects with other indications for prenatal diagnosis (controls). Placental insufficiency was assessed by histo-pathological examination. Relative measurement of telomere length was carried out prospectively by quantitative Fluorescent *In Situ* Hybridization using fluorescent Peptide Nucleic Acid probes on interphase nuclei obtained from long-term cultured villi and with an automated epifluorescent microscope. A quantitative Polymerase Chain Reaction technique was performed to confirm the quantitative Fluorescent *In Situ* Hybridization results. The number of copies of gene loci encoding the RNA template (*hTERC*) and the catalytic subunit (*hTERT*) of the enzyme complex telomerase were also estimated in these placentas by Fluorescent *In Situ* Hybridization.

**Results:**

Mean fluorescence intensity of telomere probes estimated by quantitative Fluorescent *In Situ* Hybridization was significantly less for cases compared to controls (*p*<0.001). This result indicated that mean telomere length was significantly reduced in placentas during pregnancies complicated by intrauterine growth restriction. Reduced telomere length was confirmed by the quantitative Polymerase Chain Reaction technique. No copy number variation of the *hTERC* and *hTERT* loci was noticed for cases, or for controls.

**Conclusion:**

This study clearly demonstrates a reduction of placental telomere length in ongoing pregnancies (from 18 to 37 weeks of amenorrhea) complicated by severe intrauterine growth restriction secondary to placental insufficiency.

## Introduction

Intrauterine growth restriction (IUGR) is a common complication of pregnancy which can be defined as the failure of the fetus to reach the size for which it is genetically programmed [1], [2]. In common practice, IUGR is usually detected from 20 weeks of amenorrhea (WA) onwards and is diagnosed when fetal biometrics are less than the tenth percentile [3]. Theoretically therefore, IUGR is diagnosed in 10% of pregnancies. IUGR is associated with an increase in infant mortality and with childhood and adult morbidity, with an increased risk of cardiovascular illness, obesity, and type 2 diabetes [4], [5]. The diagnosis and medical care of IUGR therefore represent a key issue for public health.

IUGR can be caused by infectious agents, chromosomal or genetic abnormalities, congenital malformations or maternal smoking; however, in the majority of cases, it is secondary to placental insufficiency [6], [7]. There are often complications with these IUGRs in the third trimester of pregnancy, with clinical-biological signs of preeclampsia [8]. The physiopathology of IUGRs associated with placental insufficiency is not fully understood, but these pathologies are usually the result of a failure of trophoblast invasion, which notably results in an intermittent blood flow of the intervillous space [1], [9], [10]. This in turn contributes locally to produce oxidative stress [10].

Telomeres are located at the ends of chromosomes comprising a repeating hexamer sequence TTAGGG and associated proteins [11], [12], [13]. Physiologically, telomeres are mainly involved in chromosome stabilization, protecting them from end-to-end fusion or degradation. The aging of somatic cells generates a gradual decrease in the length of their telomeres. Ultimately this process results in mitotic cell division coming to an end and the cell enters senescence and/or apoptosis [12], [13]. The telomere length of some cells, such as hematopoietic stem cells or germ cells, is maintained, however, thanks to the activity of the enzyme complex telomerase [14]. This enzyme consists notably of a fragment of RNA template, coded by *hTERC* (carried on locus 3q26.2), and a catalytic subunit, coded by *hTERT* (carried on locus 5p15.33). Telomere length has been assessed in different tissues and has been shown to vary from one tissue to another [12]. Apart from the influence of tissues, telomere length can also be modified by environmental factors, and it is now recognized that oxidative stress can cause a significant shortening of telomere length [15]. It has been suggested that the measurement of telomere length could be used as a biological marker for tissues suffering oxidative stress [12], [15], [16].

On the basis of all these points, two teams recently measured telomere length in placentas collected at delivery from pregnancies complicated by IUGR secondary to placental insufficiency [17], [18]. Interestingly, these authors were able to show that telomere length was significantly reduced in these placentas.

In addition, one team reported that in placentas collected at delivery from pregnancies complicated by IUGR, no copy number variations of the *hTERC* gene were observed, whereas in the controls, there were gains of the locus encoded to *hTERC* [19]. This surprising result, obtained from only a small number of placentas (5 placentas with IUGR and 5 placentas as controls), support a relative loss of *hTERC* gene copy number in placentas with IUGR that could contribute to the reduction of telomere length.

However all the above telomere length measurements had been performed in placentas collected at delivery from pregnancies complicated by IUGR and not on villus material obtained from ongoing pregnancies. The primary aim of this study was therefore to estimate placental telomere length during pregnancies complicated by severe IUGR in subjects who underwent prenatal diagnosis by late chorionic villus sampling (CVS).The secondary objective of the study was to evaluate the placental copy number of loci carrying *hTERC* and *hTERT* in a larger cohort.

## Materials and Methods

### Ethics Statement

The research protocol for this study, the relief of oral information to the patient and oral consent recording by the physician had been approved by the Bordeaux University Hospital’s Institutional Review Board. The study was based on remaining biological samples discarded after the medical routine care. All samples and data had been anonymized.

### Subjects

This prospective monocentric study was carried out between November 2010 and January 2012. Fifty-two subjects were included whose pregnancies were between 17 WA +6 days and 37 WA +2 days and for whom an invasive prenatal diagnosis was indicated. Among these subjects, 24 had severe IUGR (i.e. with fetal biometrics less than the third percentile and without cytomegalovirus infection, congenital malformation or maternal smoking) (cases). There were 28 controls for this study. These were subjects who did not have a fetus with IUGR and who had undergone prenatal diagnosis for advanced maternal age, second trimester maternal serum screening (risk >1/250 of having a child with trisomy 21), family history of chromosome or gene abnormalities (prenatal diagnosis for “antecedent”), isolated anomaly under ultrasound, and who were non-smokers (controls). As telomere length was shown to be reduced in trisomy 21 amniocytes, cases and controls were informed of the possibility of finding a cytogenetic abnormality with the Fluorescence *In Situ* Hybridization (FISH) technique to detect the main aneuploidies and/or after the conventional karyotyping and that they would be excluded from this study if so [20].

The following information was recorded: maternal age, gestational age, number of pregnancies (gravida), number of children (para), pregnancy term and birth weight, also any clinical-biological signs of preeclampsia in the course of the pregnancy (high maternal blood pressure associated with proteinuria) [21].

### Chorionic Villus Sampling

The 52 subjects in the study were offered prenatal diagnosis by late CVS. Extra-amniotic placental villi were sampled trans-abdominally by experienced physicians from our fetal medicine center [22]. The amount of placental villi sampled was greater than 30 mg for each biopsy [23].

The villi samples were then divided up to perform the following analyses: cytogenetic analysis (rapid detection of the main aneuploidies and conventional karyotyping), standard histo-pathological examination (for cases with IUGR), estimation of placental telomere length (using a quantitative FISH (Q-FISH) technique and a quantitative Polymerase Chain Reaction (Q-PCR) technique), and evaluation of copy number of the loci carrying *hTERC* and *hTERT.*

### Cytogenetic Examination

For each placental biopsy, a rapid cytogenetic examination was carried out to detect the main aneuploidies (i.e. anomalies in chromosomes 13, 18, 21 and the gonosomes) using a FISH technique (Abbott Molecular, Abbott Park, IL, USA). To ensure reliable cytogenetic results, this FISH technique was performed on cells from the mesenchymal core of the placental villi after enzymatic digestion of villi by trypsin then by collagenase [24], [25]. Conventional karyotyping was established on cultured placental villi, after Giemsa staining and heat denaturation (‘R-bands’) at a level of resolution of about 400 bands per haploid set.

### Histo-pathological Examination

A standard histo-pathological examination was carried out on the placental villi of cases with IUGR after the samples were first fixed with buffered formalin then embedded in paraffin. Sections were performed and hematoxylin-eosin-saffron staining was carried out. The main placental histo-pathological characteristics associated with IUGR secondary to placental insufficiency were examined: presence of villi that were small in size, perivillous nuclear clusters (i.e. presence of syncytial knots) and perivillous fibrin deposition [26], [27], [28], [29].

### Estimating Placental Telomere Length

After the cytogenetic and histo-pathological examinations, surplus placental villi were used to estimate placental telomere length.

About 5 mg of the placental villi were cultured on Labtek™ chamber slides (Thermo Fisher Scientific, NY, USA) [30]. After 5 to 10 days, the cells were harvested and fixed with a mixture of acetic acid and methanol (2:3, v/v). Placental telomere length in these preparations was estimated using a Q-FISH technique using Peptide Nucleic Acid (PNA) fluorescent probes. The slides were successively immersed in a solution of Tris Buffered Saline (TBS) 1X for 2 minutes, in a solution of 3.7% formaldehyde for 2 minutes, in a solution of TBS 1X for 10 minutes, in a solution of proteinase K for 10 minutes, and in a solution of TBS 1X for 10 minutes (Dako, Glostrup, Denmark). The nucleic acids were then denatured for 3 minutes in a 70% concentration formamide solution and warmed to 71°C in a water bath with thermostat control. At the same time, PNA fluorescent probes targeting the telomere sequences and stained with Cyanine 3 (Panagene, Daejeon, Korea) were denatured for 5 minutes in a water bath warmed to 81°C. The denatured probes were then placed on the Labtek™ chamber slides, and these were all put into a Hybrite™ automated co-denaturation oven (Abbott Molecular, Abbott Park, IL, USA) at 30°C for 1.5 hours to hybridize the telomere probes on the nucleic acids. The slides were then successively immersed in a rinse solution for 1 minute and in a wash solution warmed to 65°C for 5 minutes (Dako, Glostrup, Denmark). After counterstaining the nucleic acids with 4′,6′-diamidino-2-phenylindole (DAPI), the slides were placed under an Axio Imager 2 microscope with an epi-fluorescence source (Carl Zeiss AG, Oberkochen, Germany). The microscope was linked to the Metafer 4 software for automated image acquisition and processing (MetaSystems GmbH, Altlussheim, Germany). The automated microscope was programmed to acquire 1000 correctly hybridized interphase nuclei. For each of these nuclei, total telomere fluorescence intensity of PNA probes was recorded. The arithmetic mean of total telomere fluorescence intensities of PNA probes was then used for each preparation. The higher the mean telomere fluorescence intensity of PNA probes, the longer the telomere length was assumed to be. The intra-assay and inter-assay variability for the Q-FISH technique were 10.2% and 14.3%, respectively.

The remaining surplus placental villi were cultured in flasks. After 10 to 20 days, the cell cultures that had reached confluence were detached using trypsin and the cell pellets were preserved at −20°C until cellular DNA was extracted using a saline method described previously [31]. The extracted DNA was preserved at −80°C until telomere length was estimated using a Q-PCR technique, as described previously [32]. Briefly, this technique was based on telomere primers (“telg” (sense), 5′-ACACTAAGGTTTGGGTTTGGGTTTGGGTTTGGGTTAGTGT-3′; “telc” (anti-sense), 5′-TGTTAGGTATCCCTATCCCTATCCCTATCCCTATCCCTAACA-3′), primers surrounding a reference gene (in this study we used the *prostate-specific antigen* (*PSA*) gene, sense, 5′-AGGCTGGGGCAGCATTGAAC-3′; anti-sense, 5′-CACCTTCTGAGGGTGAACTTG-3′) (Eurogentec SA, Seraing, Belgium) and a Brilliant III Ultra-Fast SYBR™ Green QPCR Master Mix solution (Agilent Technologies, Santa Clara, CA, USA). Estimation of telomere length by Q-PCR was performed using the Stratagene Mx3005p system (Agilent Technologies, Santa Clara, CA, USA), according to the PCR cycles described previously [32]. Ct measurements were performed in duplicate and for each subject the mean ratio (1/Efficiency “telomere primers”^Ct “tel”^)/(1/Efficiency “PSA primers”^Ct “PSA”^) was calculated, called here the “T/S ratio”, as described previously [33]. The higher the T/S ratio, the longer the telomere length was assumed to be. The intra-assay and inter-assay variability for the Q-PCR technique were 7.24% and 11.7%, respectively.

### Copy Number of the Loci Carrying *hTERC* and *hTERT*

A copy number evaluation of the loci carrying *hTERC* (locus 3q26.2) and *hTERT* (locus 5p15.33) was performed using a FISH technique with bacterial artificial chromosome (BAC) probes RP11-816J6 (locus 3q26.2) and RP11-117B23 (locus 5p15.33), respectively (BlueGnome, Cambridge, UK). The control probes used in this study were BAC probes RP11-16N5 (locus 3q13.13) for *hTERC,* and RP11-19F12 (locus 5p13.1) for *hTERT* (BlueGnome, Cambridge, UK). The Labtek™ chamber slides used originally to estimate telomere length by the Q-FISH technique were next used to evaluate the copy number of the loci of interest. Twelve of the Labtek™ chamber slides prepared for the 24 cases with an IUGR and 12 prepared for the 28 controls were randomly selected. After denaturation of the nucleic acids and the probes for 5 minutes in 70% formamide solutions, the different mixtures from the BAC probes (“locus *hTERC* probe+control probe”; “locus *hTERT* probe+control probe”) were successively hybridized at 37°C overnight on the 24 selected slides. The Metafer 4 software was reprogrammed in order to count the number of signals from each probe. Three hundred correctly hybridized interphase nuclei were selected by the automated microscope. In these nuclei, the ratio between the number of probes of interest and the number of control probes gave the percentage of interphase nuclei showing an absence of gain or loss of the loci examined (i.e. as many signals from probes of interest as signals from control probes), the percentage showing relative losses of the locus of interest (e.g. two signals from the locus of interest and three signals from the control locus), the percentage showing relative gains of the locus of interest (e.g. two signals from the locus of interest and one signal from the control locus) and the percentage showing absolute gains of the locus of interest (i.e. three signals or more from the locus of interest and two signals from the control locus).

### Statistics

Distributions of the Q-FISH and Q-PCR measurements were tested for normality with the Shapiro-Wilk test. Univariate analyses of quantitative variables were performed using either the Student’s t-test, the Wilcoxon-Mann Whitney test or the Pearson’s correlation test, whichever was appropriate. A multivariate analysis was carried out using a logistic regression to estimate the influence of certain covariates (maternal age, number of pregnancies, number of children and term of placental biopsy) on the mean telomere fluorescence intensity of PNA probes observed with the Q-FISH technique. The significance level was set at an α level of 0.05 and STATA 8.0 (StataCorp LP, College Station, TX, USA) and R (www.r-project.org) softwares were used for statistical analyses.

## Results

### Subjects, Cytogenetic and Histo-pathological Examinations

The characteristics of the subjects and their pregnancies are summarized in Table 1. Maternal age was significantly lower for cases (29±7, mean ± standard deviation (SD), years) compared to controls (34±7, mean ± SD, years) (*p*<0.05), probably due to the high number of controls who had performed prenatal diagnosis for advance maternal age (10/28 = 35.7%) (Table 1). There was no statistically significant difference in number of pregnancies (gravida), number of children (para) and term of placental biopsy between cases with an IUGR and controls (*p* = non-significant (NS), Student’s t-test or Wilcoxon-Mann Whitney test) (Table 1). Cytogenetic analysis (rapid detection of the main aneuploidies by FISH and conventional karyotyping) did not show any fetal chromosomal abnormalities in the subjects of the study. In 21 of the 24 placentas of cases with IUGR (87.5%), standard histo-pathological examination of the placental villi showed morphological characteristics compatible with an IUGR secondary to placental insufficiency. As expected, birth term was earlier and birth weight was lower in cases with an IUGR compared with the controls (*p*<0.01, Student’s t-test) (Table 1). Of the 24 cases with an IUGR, 3 (12.5%) presented clinical-biological signs of preeclampsia during their pregnancy. None of the controls developed clinical-biological signs of preeclampsia.

**Table 1:** Main characteristics of the subjects and their pregnancies.

Characteristics	Controls (n = 28)	IUGR (n = 24)	p-value
Indication for prenatal diagnosis (n, %)	Advanced maternal age (10/28, 35.7)	Severe IUGR (24/24, 100)	
	Second trimester MSS (4/28, 14.3)		
	Antecedent (3/28, 10.7)		
	US anomaly (11/28, 39.3)		
Maternal age (years) (mean ± SD)	34±7	29±7	<0.05
Gravida (n) (median (Q1–Q3))	3 (2–3)	2 (1–3)	NS
Para (n) (median (Q1–Q3))	1 (0–2)	1 (0–2)	NS
CVS term (WA) (mean ± SD)	27±6	29±5	NS
Birth term (WA) (mean ± SD)	39±2	36±2	<0.01
Birth weight (g) (mean ± SD)	3299±719	1932±458	<0.001
Telomere fluorescence intensity (AU) (mean ± SD)	5166±1003	4336±553	<0.001

AU: arbitrary units; IUGR; intrauterine growth restriction; MSS; second trimester maternal serum screening; US: ultrasound.

### Estimation of Placental Telomere Length

**Figure 1:**
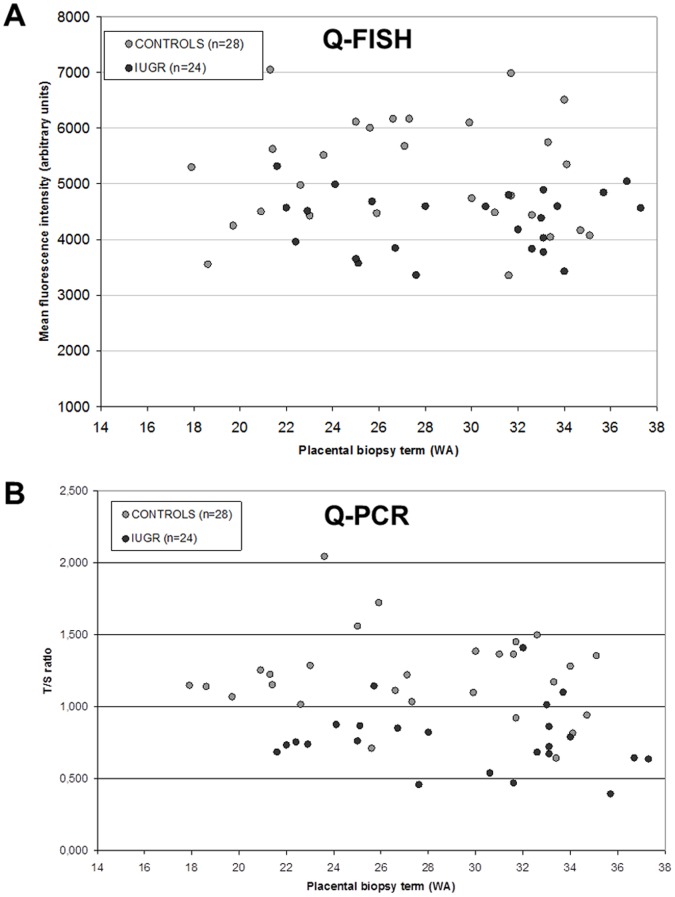
Placental telomere length estimated from late chorionic villus samplings by two quantitative techniques. A. Quantitative Fluorescence *In Situ* Hybridization (Q-FISH). Telomere fluorescence intensity of Peptide Nucleic Acid probes (arbitrary units) as a function of pregnancy term of placental biopsy (expressed in weeks of amenorrhea (WA)) for the 28 controls (light grey circles) and the 24 cases with intrauterine growth restriction (IUGR) (dark grey circles). B. Quantitative Polymerase Chain Reaction (Q-PCR). T/S ratios (no unit) as a function of pregnancy term of placental biopsy (expressed in WA) for the 28 controls (light grey circles) and the 24 cases with IUGR (dark grey circles).

**Figure 2:**
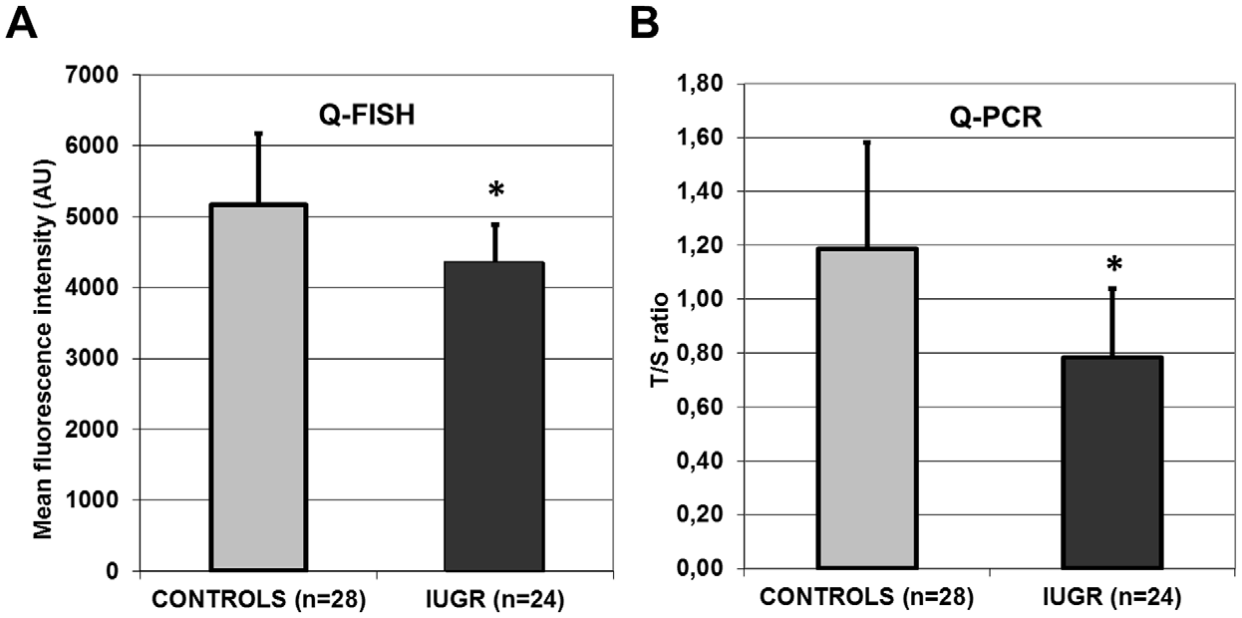
Comparison of placental telomere length between controls and cases with intrauterine growth restriction (IUGR), with two quantitative techniques. A. Quantitative Fluorescence *In Situ* Hybridization (Q-FISH). Mean placental telomere fluorescence intensity of Peptide Nucleic Acid probes with the Q-FISH technique ± standard deviation (arbitrary units) for the 28 controls (light grey histogram) and the 24 cases with IUGR (dark grey histogram) (**: *p*<0.001, Student’s t-test). B. Quantitative Polymerase Chain Reaction (Q-PCR). Mean placental telomere T/S ratio with the Q-PCR technique ± standard deviation (no unit) for the 28 controls (light grey histogram) and the 24 cases with IUGR (dark grey histogram) (**: *p*<0.001, Student’s t-test).

Figure 1A illustrates the telomere fluorescence intensities of PNA probes as a function of pregnancy term of placental biopsy for the cases with IUGR and the controls (Q-FISH technique). The placental telomere fluorescence intensities followed a normal distribution (*p* = NS, Shapiro-Wilk test). The mean placental telomere fluorescence intensity of PNA probes was less in the cases with an IUGR (4336±553, mean ± SD, arbitrary units) compared with the controls (5166±1003, mean ± SD, arbitrary units) (*p*<0.001, Student’s t-test) (Figure 2A). The mean telomere fluorescence intensity of PNA probes was thus 16.1% less in the placentas of cases with an IUGR. Multivariate analysis by logistic regression (adjustment for maternal age, number of pregnancies, number of children, and term of placental biopsy) confirmed the decrease in placental telomere fluorescence intensity in cases with an IUGR (*p*<0.001). Multivariate analysis also revealed that the covariates selected (maternal age, number of pregnancies, number of children and term of placental biopsy) had no influence on placental telomere length (*p* = NS). The results of the multivariate analysis could be interpreted as follows, a decrease of 1000 arbitrary units of placental telomere fluorescence with the Q-FISH technique would predispose to a 4 times higher risk of severe IUGR.

Figure 1B illustrates the T/S ratios as a function of pregnancy term of placental biopsy for the cases with IUGR and the controls (Q-PCR technique). The T/S ratios followed a normal distribution (*p* = NS, Shapiro-Wilk test). The Q-PCR technique confirmed that mean placental telomere length was reduced in the cases with an IUGR (ratio T/S: 0.78±0.26, mean ± SD, no unit) compared with the controls (T/S ratio: 1.19±0.40, mean ± SD, no unit) (*p*<0.001, Student’s t-test) (Figure 2B). The mean T/S ratio was therefore 34.5% less in the placentas of the cases with an IUGR. Q-PCR and Q-FISH results were significantly associated (*p* = 0.026, Pearson’s correlation test).

Regarding the term of placental biopsy, no influence on placental telomere fluorescence intensity or T/S ratios was observed for either cases with IUGR, controls or all subjects in this study (cases+controls) (*p* = NS, Pearson’s correlation test).

### Copy Number of the Loci Carrying *hTERC* and *hTERT*

**Figure 3:**
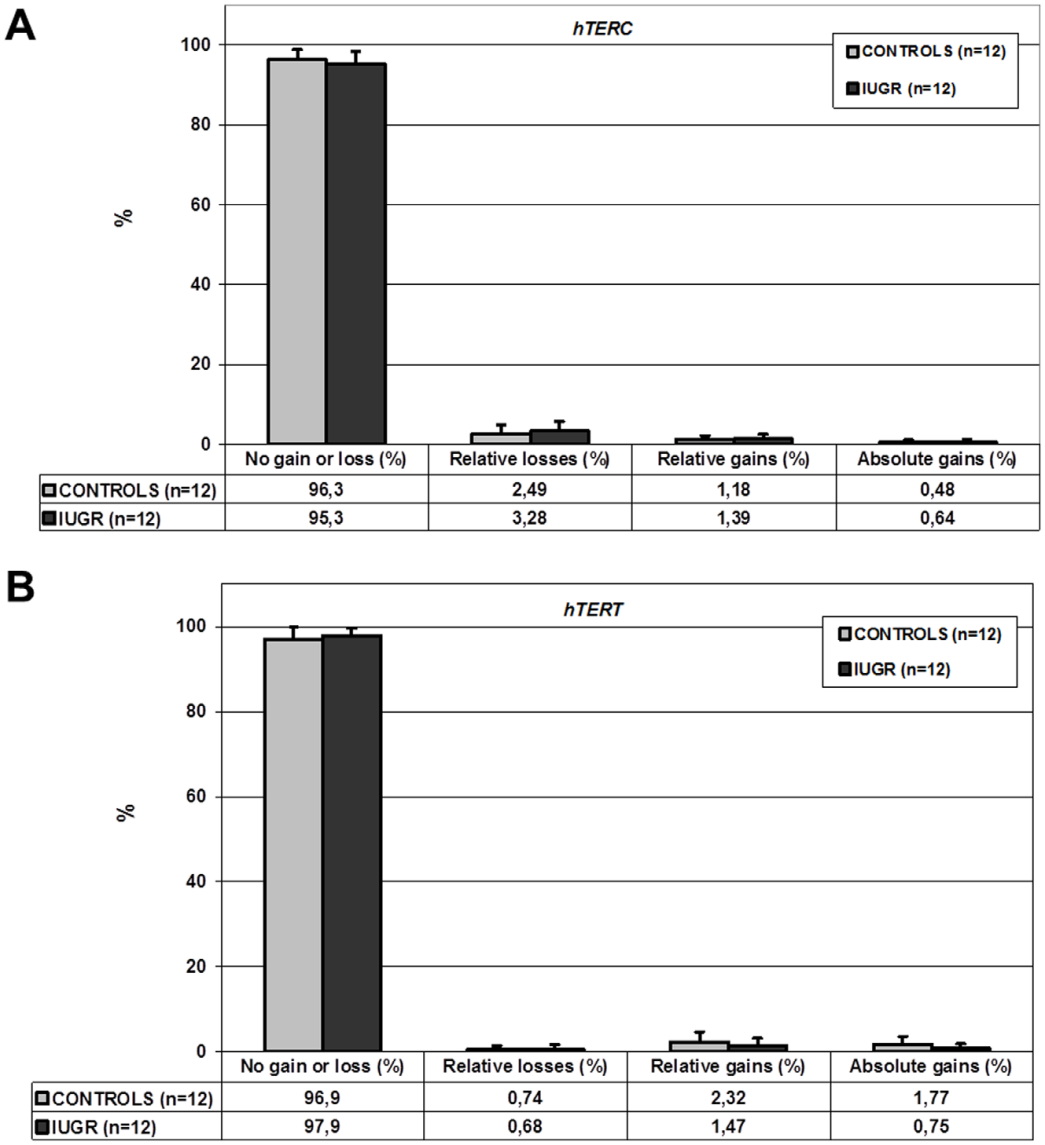
Copy number evaluation by Fluorescence *In Situ* Hybridization (FISH) of the loci carrying *hTERC* and *hTERT* in late chorionic villus samplings. A. Percentage of interphase nuclei displaying no gain or loss, relative losses, relative gains, and absolute gains of *hTERC* locus with the FISH technique ± standard deviation (%) for 12 controls (light grey histograms) and 12 cases with intrauterine growth restriction (IUGR) (dark grey histograms) randomly selected (*p* = non-significant (NS), Student’s t-test). B. Percentage of interphase nuclei displaying no gain or loss, relative losses, relative gains, and absolute gains of *hTERT* locus with the FISH technique ± standard deviation (%) for 12 controls (light grey histograms) and 12 cases with IUGR (dark grey histograms) randomly selected (*p* = NS, Student’s t-test).

There was no difference in the hybridization profiles of the BAC probe targeting the locus carrying *hTERC* between cases with an IUGR and the controls (*p* = NS, Wilcoxon-Mann Whitney test); in particular, no gains of this locus were observed in the 24 placentas studied (Figure 3A). The same was true for the BAC probe targeting the locus carrying *hTERT* (*p* = NS, Wilcoxon-Mann Whitney test) (Figure 3B).

## Discussion

In our fetal medicine center, more than 80% of our prenatal diagnoses consist of early and late CVS, rather than amniocentesis, whatever the pregnancy term [22], [34]. For 30 years, we have therefore performed more than 26,000 early and late CVS [22]. These procedures have notably the advantage of providing a conventional karyotype after 6–8 days culture, which is quicker than with amniocentesis [22]. In addition, we propose a prenatal diagnosis by late CVS to patients with an idiopathic IUGR, rather than by amniocentesis, the aim being, on the one hand, to look for a confined placental mosaicism which could explain the fetal growth restriction and, on the other hand, to perform a standard histo-pathological examination to provide arguments supporting a placental origin of the IUGR [27], [30].

The Q-FISH technique was used successfully on all the Labtek™ chamber slides prepared from the placental biopsies. It showed that mean placental telomere length was reduced between 18 and 37 WA in pregnancies with severe IUGR. Gestational age at the term of placental biopsy had no influence on telomere length. This result could notably suggest that the reduction of placental telomere length takes place early in the pregnancies with an IUGR secondary to placental insufficiency. Reduced placental telomere length does not appear to be only a late phenomenon, appearing towards the end of pregnancies with an IUGR, as initially reported [17], [18].

Results from placental telomere length estimation using the Q-FISH technique were confirmed by the Q-PCR technique. It was interesting to note that the difference in mean telomere length between the cases with IUGR and the controls was much greater with Q-PCR (mean difference 34.5%) than with Q-FISH (mean difference 16.1%), which seems to indicate that the Q-PCR technique is probably more sensitive for estimating telomere length than the Q-FISH technique. Indeed, the Q-PCR technique that we used had been described previously as having some good characteristics and it had also been suggested that estimating telomere length by an automated Q-FISH technique could be biased by the auto-fluorescence of preparations [11], [35]. This could account for the lower performance of the Q-FISH technique in showing the difference in telomere length between our cases with IUGR and our controls. In future, it will therefore probably be advisable to prefer the Q-PCR technique that we used here to estimate placental telomere length.

We observed no placental copy number variation of the locus carrying *hTERC* in our cases with IUGR and in our controls. This result are at clear variance with those reported by Biron-Shental *et* *al.* who stated that the number of copies of the locus carrying *hTERC* was less in placentas collected at delivery from pregnancies complicated by an IUGR secondary to placental insufficiency [19]. Indeed, they observed that gains of this locus were present in over 8% of interphase nuclei in placentas at delivery in their controls, whereas these gains were only found in about 3% of interphase nuclei in placentas collected at delivery from their cases with IUGR. The authors then hypothesized that *hTERC* gains could be a physiological process that takes place in the placenta and that such phenomenon could be altered in pregnancies with an IUGR. However, these authors studied 5 placental villi collected at delivery from pregnancies with an IUGR and 5 collected at delivery from control pregnancies. In our study of a larger cohort (12 samples for each group), the number of signals from BAC probes was automatically counted from 300 interphase nuclei. With this method we were unable to confirm the presence of *hTERC* gains in the placenta during our control pregnancies and visual inspection of our slides did not also detect such phenomemon.

We also aimed at determining the copy number of the locus carrying *hTERT* gene. Indeed, it has been shown that TERT expression is closely correlated with the presence of telomerase activity in the placenta [36]. In patients with an IUGR, some authors have noted an absence of telomerase activity in the placenta related to reduced TERT expression, whereas telomerase activity and TERT expression were maintained in placentas in pregnancies without complications [17], [18], [37]. TERT expression would be a limiting factor on telomerase activity at placental level, and as a result, its lower expression could account for the reduction of telomere length that we report during pregnancies with an IUGR secondary to placental insufficiency. Although we have not considered TERT expression here, our study did not find any placental copy number variation affecting the locus carrying the *hTERT* gene. The reduced TERT expression in the placenta is likely linked with a down-regulation of *hTERT* or with alternative RNA splicing of transcripts of this gene in cases of IUGR [37].

In this study we have only estimated telomere length in the placenta. It would be interesting also to study telomere length in amniocytes during pregnancies complicated by an IUGR. Davy *et al.* demonstrated that, at delivery, cord blood cell telomere length was identical in pregnancies with an IUGR and in control pregnancies, whereas placental telomere length was reduced in the cases with IUGR [17]. Based on such results, one would hypothesize that telomere length would not be reduced in amniocytes during pregnancies complicated by IUGR but such fact remains to be determined.

In summary, this study is the first to show that placental telomere length is reduced during pregnancy in patients with a severe IUGR secondary to placental insufficiency, and that this is the case from 18 to 37 weeks of amenorrhea. This placental telomere reduction in pregnancies with IUGR is consistent with studies that identified oxidative stress and a lack of telomerase activity in these placentas [10], [18], [37]. The absence of this activity is likely to be explained by a decrease in the expression of TERT [18], [19], [36], [37]. Therefore, telomerase activity together with TERT expression has to be investigated in such placentas in order to evaluate the usefulness of these markers in the pathophysiology and the diagnosis of IUGR.
